# [1-(4-Hydr­oxy-2-oxidobenzyl­idene)-4-phenyl­thio­semicarbazonato-κ^3^ *N*,*O*,*S*](1,10-phenanthroline-κ^2^ *N*,*N*′)zinc(II)–4,4′-bipyridine (2/1)

**DOI:** 10.1107/S1600536809026245

**Published:** 2009-07-15

**Authors:** Kong Wai Tan, Chew Hee Ng, Mohd Jamil Maah, Seik Weng Ng

**Affiliations:** a Department of Chemistry, University of Malaya, 50603 Kuala Lumpur, Malaysia; b Faculty of Engineering and Science, Universiti Tunku Abdul Rahman, 53300 Kuala Lumpur, Malaysia

## Abstract

The Zn^II^ atom in the title compound, [Zn(C_14_H_11_N_3_O_2_S)(C_12_H_8_N_2_)]·0.5C_10_H_8_N_2_, is *N*,*N*′-chelated by the *N*-heterocycle and *N*,*O*,*S*-chelated by the deprotonated Schiff base in a square-pyramidal environment. The hydr­oxy group of the Schiff base is a hydrogen-bond donor to 4,4′-bipyridine, which is located about a center of inversion, resulting in the formation of a supra­molecular trimeric unit.

## Related literature

For [1-(4-hydr­oxy-2-oxidobenzyl­idene)-4-phenyl­thio­semi­car­bazon­ato](1,10-phenanthroline)zinc dimethyl sulfoxide disolvate hydrate, see: Tan *et al.* (2009 ▶). For other *N*-heterocyclic adducts of zinc 1-(2-oxidobenzyl­idene)-4-phenyl­thio­semi­carbonates, see: Deng *et al.* (2007 ▶); Seena & Kurup (2008 ▶).
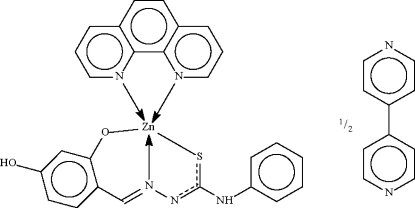


## Experimental

### 

#### Crystal data

[Zn(C_14_H_11_N_3_O_2_S)(C_12_H_8_N_2_)]·0.5C_10_H_8_N_2_
*M* *_r_* = 608.98
Monoclinic, 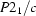

*a* = 11.6372 (6) Å
*b* = 9.8376 (5) Å
*c* = 23.288 (1) Å
β = 92.417 (3)°
*V* = 2663.7 (2) Å^3^
*Z* = 4
Mo *K*α radiation
μ = 1.04 mm^−1^
*T* = 140 K
0.10 × 0.04 × 0.02 mm

#### Data collection

Bruker SMART APEX diffractometer
Absorption correction: multi-scan (*SADABS*; Sheldrick, 1996 ▶) *T*_min_ = 0.903, *T*_max_ = 0.979
14867 measured reflections
4681 independent reflections
2918 reflections with *I* > 2σ(*I*)
*R*_int_ = 0.089

#### Refinement

*R*[*F* ^2^ > 2σ(*F* ^2^)] = 0.047
*wR*(*F* ^2^) = 0.117
*S* = 0.98
4681 reflections
378 parameters
2 restraints
H atoms treated by a mixture of independent and constrained refinement
Δρ_max_ = 0.38 e Å^−3^
Δρ_min_ = −0.40 e Å^−3^

### 

Data collection: *APEX2* (Bruker, 2008 ▶); cell refinement: *SAINT* (Bruker, 2008 ▶); data reduction: *SAINT*; program(s) used to solve structure: *SHELXS97* (Sheldrick, 2008 ▶); program(s) used to refine structure: *SHELXL97* (Sheldrick, 2008 ▶); molecular graphics: *X-SEED* (Barbour, 2001 ▶); software used to prepare material for publication: *publCIF* (Westrip, 2009 ▶).

## Supplementary Material

Crystal structure: contains datablocks I, global. DOI: 10.1107/S1600536809026245/tk2484sup1.cif

Structure factors: contains datablocks I. DOI: 10.1107/S1600536809026245/tk2484Isup2.hkl

Additional supplementary materials:  crystallographic information; 3D view; checkCIF report

## Figures and Tables

**Table 1:** Hydrogen-bond geometry (Å, °)

*D*—H⋯*A*	*D*—H	H⋯*A*	*D*⋯*A*	*D*—H⋯*A*
O2—H2⋯N6	0.84 (5)	2.01 (5)	2.839 (6)	168 (6)

**Fig. 1.**
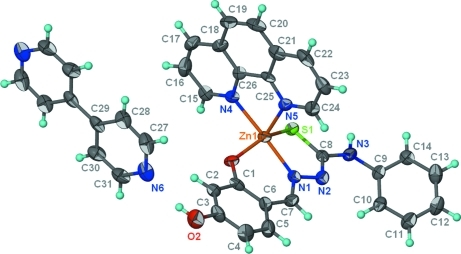
Thermal ellipsoid (Barbour, 2001) plot of Zn(C12H8N2)(C14H11N3O2S).0.5C10H8N2 at the 70% probability level. Only atoms comprising the asymmetric unit are labelled; the 4,4'-bipyridine molecule is located about a center of inversion. Hydrogen atoms are drawn as spheres of arbitrary radii.

## supplementary crystallographic information

### Experimental

Zinc acetate monohydrate (0.22 g, 1 mmol), 2,4-dihydroxybenzaldehyde 4-phenylthiosemicarbazone (0.29 g, 1 mmol) and 1,10-phenanthroline (0.18 g, 1 mmol) were heated in ethanol (50 ml). The product was isolated and reacted with 4,4'-bipyridine (0.15 g, 1 mmol) in DMF to give a yellow solution. A small amount of tiny crystals were isolated when the mixture was set aside for a week.

### Refinement

Carbon-bound H-atoms were placed in calculated positions (C—H 0.95 Å) and were included in the refinement in the riding model approximation with *U*(H) set to 1.2*U*_eq_(C). The amino- and hydroxy H-atoms were refined with distance restraints of O–H 0.84±0.01 Å and N–H 0.88±0.01 Å, respectively; their temperature factors were refined.

### Crystal data

**Table:**

[Zn(C_14_H_11_N_3_O_2_S)(C_12_H_8_N_2_)]·0.5C_10_H_8_N_2_	*F*(000) = 1252
*M**_r_* = 608.98	*D*_x_ = 1.519 Mg m^−^^3^
Monoclinic, *P*2_1_/*c*	Mo *K*α radiation, λ = 0.71073 Å
Hall symbol: -P 2ybc	Cell parameters from 1335 reflections
*a* = 11.6372 (6) Å	θ = 2.4–20.2°
*b* = 9.8376 (5) Å	µ = 1.04 mm^−^^1^
*c* = 23.288 (1) Å	*T* = 140 K
β = 92.417 (3)°	Prism, yellow
*V* = 2663.7 (2) Å^3^	0.10 × 0.04 × 0.02 mm
*Z* = 4	

### Data collection

**Table:**

Bruker SMART APEX diffractometer	4681 independent reflections
Radiation source: fine-focus sealed tube	2918 reflections with *I* > 2σ(*I*)
graphite	*R*_int_ = 0.089
ω scans	θ_max_ = 25.0°, θ_min_ = 1.8°
Absorption correction: multi-scan (*SADABS*; Sheldrick, 1996)	*h* = −13→13
*T*_min_ = 0.903, *T*_max_ = 0.979	*k* = −11→11
14867 measured reflections	*l* = −27→27

### Refinement

**Table:**

Refinement on *F*^2^	Primary atom site location: structure-invariant direct methods
Least-squares matrix: full	Secondary atom site location: difference Fourier map
*R*[*F*^2^ > 2σ(*F*^2^)] = 0.047	Hydrogen site location: inferred from neighbouring sites
*wR*(*F*^2^) = 0.117	H atoms treated by a mixture of independent and constrained refinement
*S* = 0.98	*w* = 1/[σ^2^(*F*_o_^2^) + (0.0515*P*)^2^] where *P* = (*F*_o_^2^ + 2*F*_c_^2^)/3
4681 reflections	(Δ/σ)_max_ = 0.001
378 parameters	Δρ_max_ = 0.38 e Å^−^^3^
2 restraints	Δρ_min_ = −0.40 e Å^−^^3^

### Fractional atomic coordinates and isotropic or equivalent isotropic displacement parameters (Å^2^)

**Table:**

	*x*	*y*	*z*	*U*_iso_*/*U*_eq_	
Zn1	0.67107 (4)	0.62440 (5)	0.56348 (2)	0.02152 (16)	
S1	0.82614 (10)	0.50702 (12)	0.52318 (5)	0.0236 (3)	
O1	0.5106 (2)	0.6211 (3)	0.58865 (12)	0.0257 (7)	
O2	0.2250 (3)	0.7085 (4)	0.72356 (16)	0.0416 (9)	
H2	0.188 (5)	0.738 (6)	0.6945 (16)	0.07 (2)*	
N1	0.7189 (3)	0.5107 (4)	0.63475 (15)	0.0216 (9)	
N2	0.8294 (3)	0.4544 (4)	0.63924 (15)	0.0256 (9)	
N3	0.9923 (3)	0.4067 (4)	0.59142 (16)	0.0263 (10)	
H3	1.029 (3)	0.427 (5)	0.5604 (12)	0.036 (15)*	
N4	0.6154 (3)	0.7367 (4)	0.48877 (15)	0.0218 (9)	
N5	0.7320 (3)	0.8198 (4)	0.58516 (15)	0.0222 (9)	
N6	0.1202 (3)	0.8459 (4)	0.62781 (18)	0.0371 (11)	
C1	0.4781 (4)	0.6105 (5)	0.64195 (18)	0.0242 (10)	
C2	0.3686 (4)	0.6607 (5)	0.6553 (2)	0.0272 (12)	
H2A	0.3205	0.6984	0.6254	0.033*	
C3	0.3294 (4)	0.6565 (5)	0.7103 (2)	0.0327 (13)	
C4	0.3969 (4)	0.5966 (5)	0.7542 (2)	0.0380 (14)	
H4	0.3703	0.5929	0.7922	0.046*	
C5	0.5007 (4)	0.5436 (6)	0.7423 (2)	0.0364 (13)	
H5A	0.5445	0.5004	0.7724	0.044*	
C6	0.5469 (4)	0.5492 (5)	0.68749 (19)	0.0261 (11)	
C7	0.6604 (4)	0.4965 (5)	0.68069 (19)	0.0259 (11)	
H7	0.6954	0.4477	0.7120	0.031*	
C8	0.8810 (4)	0.4554 (4)	0.59072 (19)	0.0217 (11)	
C9	1.0617 (4)	0.3594 (5)	0.63889 (19)	0.0237 (11)	
C10	1.0185 (4)	0.2924 (5)	0.68556 (19)	0.0281 (12)	
H10	0.9378	0.2823	0.6887	0.034*	
C11	1.0937 (4)	0.2399 (5)	0.7278 (2)	0.0353 (13)	
H11	1.0642	0.1921	0.7594	0.042*	
C12	1.2121 (4)	0.2566 (5)	0.7242 (2)	0.0365 (13)	
H12	1.2633	0.2211	0.7533	0.044*	
C13	1.2540 (4)	0.3249 (5)	0.6781 (2)	0.0376 (14)	
H13	1.3346	0.3377	0.6757	0.045*	
C14	1.1812 (4)	0.3749 (5)	0.63541 (19)	0.0276 (11)	
H14	1.2116	0.4202	0.6034	0.033*	
C15	0.5563 (4)	0.6950 (5)	0.44181 (19)	0.0253 (11)	
H15	0.5412	0.6006	0.4374	0.030*	
C16	0.5157 (4)	0.7832 (6)	0.3990 (2)	0.0365 (14)	
H16	0.4727	0.7499	0.3664	0.044*	
C17	0.5390 (4)	0.9194 (6)	0.4048 (2)	0.0331 (13)	
H17	0.5125	0.9813	0.3759	0.040*	
C18	0.6018 (4)	0.9676 (5)	0.45336 (19)	0.0281 (12)	
C19	0.6260 (4)	1.1078 (5)	0.4628 (2)	0.0367 (13)	
H19	0.5970	1.1731	0.4359	0.044*	
C20	0.6895 (4)	1.1493 (5)	0.5093 (2)	0.0380 (14)	
H20	0.7080	1.2429	0.5136	0.046*	
C21	0.7297 (4)	1.0533 (5)	0.5525 (2)	0.0307 (12)	
C22	0.7924 (4)	1.0906 (5)	0.6035 (2)	0.0345 (13)	
H22	0.8121	1.1830	0.6104	0.041*	
C23	0.8244 (4)	0.9928 (5)	0.6427 (2)	0.0339 (13)	
H23	0.8681	1.0161	0.6767	0.041*	
C24	0.7919 (4)	0.8584 (5)	0.63216 (19)	0.0294 (12)	
H24	0.8137	0.7912	0.6599	0.035*	
C25	0.7022 (4)	0.9150 (4)	0.54532 (19)	0.0202 (10)	
C26	0.6384 (4)	0.8709 (5)	0.49423 (18)	0.0212 (10)	
C27	0.1329 (4)	0.9783 (5)	0.6168 (2)	0.0398 (14)	
H27	0.1749	1.0319	0.6443	0.048*	
C28	0.0887 (4)	1.0418 (5)	0.5679 (2)	0.0343 (13)	
H28	0.1017	1.1361	0.5624	0.041*	
C29	0.0256 (4)	0.9682 (5)	0.52677 (19)	0.0281 (12)	
C30	0.0133 (5)	0.8298 (5)	0.5379 (2)	0.0347 (13)	
H30	−0.0280	0.7736	0.5110	0.042*	
C31	0.0606 (4)	0.7748 (5)	0.5876 (2)	0.0378 (13)	
H31	0.0504	0.6802	0.5938	0.045*	

### Atomic displacement parameters (Å^2^)

**Table:**

	*U*^11^	*U*^22^	*U*^33^	*U*^12^	*U*^13^	*U*^23^
Zn1	0.0209 (3)	0.0191 (3)	0.0250 (3)	0.0013 (3)	0.0065 (2)	0.0033 (3)
S1	0.0228 (7)	0.0254 (7)	0.0230 (6)	0.0049 (5)	0.0050 (5)	0.0021 (5)
O1	0.0252 (18)	0.0252 (17)	0.0272 (17)	0.0055 (16)	0.0083 (14)	0.0099 (16)
O2	0.028 (2)	0.058 (3)	0.041 (2)	0.0082 (19)	0.0113 (19)	−0.001 (2)
N1	0.019 (2)	0.020 (2)	0.026 (2)	0.0015 (17)	0.0051 (17)	−0.0013 (17)
N2	0.022 (2)	0.029 (2)	0.026 (2)	0.0051 (18)	0.0033 (18)	0.0001 (18)
N3	0.025 (2)	0.033 (3)	0.022 (2)	0.0056 (19)	0.0056 (19)	0.0049 (19)
N4	0.024 (2)	0.020 (2)	0.022 (2)	0.0007 (17)	0.0064 (17)	−0.0015 (17)
N5	0.022 (2)	0.025 (2)	0.020 (2)	0.0028 (17)	0.0074 (18)	0.0015 (17)
N6	0.024 (2)	0.044 (3)	0.044 (3)	0.005 (2)	0.010 (2)	−0.012 (2)
C1	0.022 (3)	0.023 (3)	0.028 (3)	−0.006 (2)	0.011 (2)	−0.005 (2)
C2	0.021 (3)	0.033 (3)	0.028 (3)	−0.001 (2)	0.006 (2)	0.004 (2)
C3	0.018 (3)	0.044 (3)	0.037 (3)	−0.002 (2)	0.009 (2)	−0.005 (2)
C4	0.032 (3)	0.062 (4)	0.020 (3)	0.000 (3)	0.009 (2)	−0.003 (3)
C5	0.025 (3)	0.058 (4)	0.027 (3)	0.000 (3)	0.001 (2)	−0.001 (3)
C6	0.022 (3)	0.035 (3)	0.022 (2)	−0.003 (2)	0.002 (2)	−0.004 (2)
C7	0.029 (3)	0.029 (3)	0.020 (2)	0.001 (2)	0.004 (2)	0.004 (2)
C8	0.020 (3)	0.017 (3)	0.028 (3)	0.000 (2)	0.003 (2)	0.000 (2)
C9	0.024 (3)	0.023 (3)	0.025 (2)	0.008 (2)	0.001 (2)	−0.002 (2)
C10	0.027 (3)	0.026 (3)	0.031 (3)	0.007 (2)	−0.003 (2)	0.002 (2)
C11	0.036 (3)	0.043 (3)	0.027 (3)	0.010 (3)	0.001 (2)	0.002 (3)
C12	0.032 (3)	0.051 (4)	0.026 (3)	0.013 (3)	−0.002 (2)	−0.002 (3)
C13	0.026 (3)	0.050 (4)	0.036 (3)	0.007 (3)	−0.001 (3)	−0.009 (3)
C14	0.027 (3)	0.028 (3)	0.028 (2)	0.001 (2)	0.005 (2)	−0.003 (2)
C15	0.024 (3)	0.026 (3)	0.026 (3)	0.002 (2)	0.007 (2)	−0.007 (2)
C16	0.027 (3)	0.064 (4)	0.019 (3)	0.008 (3)	0.001 (2)	−0.006 (3)
C17	0.028 (3)	0.050 (4)	0.022 (3)	0.013 (3)	0.010 (2)	0.012 (2)
C18	0.027 (3)	0.032 (3)	0.027 (3)	0.009 (2)	0.012 (2)	0.008 (2)
C19	0.025 (3)	0.033 (3)	0.052 (3)	0.010 (3)	0.015 (3)	0.021 (3)
C20	0.028 (3)	0.021 (3)	0.067 (4)	0.005 (2)	0.022 (3)	0.009 (3)
C21	0.024 (3)	0.029 (3)	0.040 (3)	0.002 (2)	0.016 (2)	−0.005 (3)
C22	0.024 (3)	0.029 (3)	0.052 (3)	−0.007 (2)	0.015 (3)	−0.016 (3)
C23	0.028 (3)	0.037 (3)	0.037 (3)	−0.009 (3)	0.005 (2)	−0.015 (3)
C24	0.027 (3)	0.037 (3)	0.025 (3)	0.000 (2)	0.006 (2)	−0.001 (2)
C25	0.017 (2)	0.014 (2)	0.030 (3)	−0.0016 (19)	0.012 (2)	−0.001 (2)
C26	0.018 (2)	0.020 (2)	0.026 (2)	0.007 (2)	0.0075 (19)	−0.001 (2)
C27	0.028 (3)	0.038 (3)	0.053 (4)	0.002 (3)	−0.002 (3)	−0.022 (3)
C28	0.032 (3)	0.024 (3)	0.047 (3)	0.002 (2)	0.002 (3)	−0.012 (3)
C29	0.018 (3)	0.029 (3)	0.038 (3)	−0.003 (2)	0.011 (2)	−0.012 (2)
C30	0.039 (3)	0.026 (3)	0.041 (3)	−0.006 (2)	0.009 (3)	−0.007 (2)
C31	0.035 (3)	0.028 (3)	0.051 (3)	−0.007 (3)	0.011 (3)	−0.003 (3)

### Geometric parameters (Å, °)

**Table:**

Zn1—O1	1.981 (3)	C11—C12	1.394 (7)
Zn1—N1	2.058 (4)	C11—H11	0.9500
Zn1—N5	2.103 (4)	C12—C13	1.373 (7)
Zn1—N4	2.138 (3)	C12—H12	0.9500
Zn1—S1	2.3692 (12)	C13—C14	1.370 (6)
S1—C8	1.748 (4)	C13—H13	0.9500
O1—C1	1.317 (5)	C14—H14	0.9500
O2—C3	1.365 (6)	C15—C16	1.389 (6)
O2—H2	0.84 (5)	C15—H15	0.9500
N1—C7	1.299 (5)	C16—C17	1.373 (7)
N1—N2	1.401 (5)	C16—H16	0.9500
N2—C8	1.302 (5)	C17—C18	1.402 (7)
N3—C8	1.380 (6)	C17—H17	0.9500
N3—C9	1.419 (6)	C18—C26	1.399 (6)
N3—H3	0.88 (3)	C18—C19	1.423 (7)
N4—C15	1.332 (5)	C19—C20	1.347 (7)
N4—C26	1.352 (6)	C19—H19	0.9500
N5—C24	1.328 (5)	C20—C21	1.445 (7)
N5—C25	1.353 (5)	C20—H20	0.9500
N6—C27	1.336 (7)	C21—C25	1.406 (6)
N6—C31	1.340 (6)	C21—C22	1.414 (7)
C1—C2	1.413 (6)	C22—C23	1.368 (7)
C1—C6	1.434 (6)	C22—H22	0.9500
C2—C3	1.379 (6)	C23—C24	1.394 (7)
C2—H2A	0.9500	C23—H23	0.9500
C3—C4	1.392 (7)	C24—H24	0.9500
C4—C5	1.356 (7)	C25—C26	1.442 (6)
C4—H4	0.9500	C27—C28	1.379 (7)
C5—C6	1.406 (6)	C27—H27	0.9500
C5—H5A	0.9500	C28—C29	1.386 (6)
C6—C7	1.435 (6)	C28—H28	0.9500
C7—H7	0.9500	C29—C30	1.394 (7)
C9—C10	1.384 (6)	C29—C29^i^	1.495 (9)
C9—C14	1.405 (6)	C30—C31	1.371 (7)
C10—C11	1.388 (6)	C30—H30	0.9500
C10—H10	0.9500	C31—H31	0.9500
			
O1—Zn1—N1	88.77 (13)	C13—C12—C11	119.3 (5)
O1—Zn1—N5	104.84 (13)	C13—C12—H12	120.3
N1—Zn1—N5	103.07 (14)	C11—C12—H12	120.3
O1—Zn1—N4	89.56 (13)	C14—C13—C12	120.9 (5)
N1—Zn1—N4	177.58 (14)	C14—C13—H13	119.6
N5—Zn1—N4	79.07 (14)	C12—C13—H13	119.6
O1—Zn1—S1	148.49 (10)	C13—C14—C9	120.2 (5)
N1—Zn1—S1	82.54 (10)	C13—C14—H14	119.9
N5—Zn1—S1	106.62 (10)	C9—C14—H14	119.9
N4—Zn1—S1	98.00 (10)	N4—C15—C16	123.0 (5)
C8—S1—Zn1	92.34 (15)	N4—C15—H15	118.5
C1—O1—Zn1	126.3 (3)	C16—C15—H15	118.5
C3—O2—H2	113 (4)	C17—C16—C15	118.6 (5)
C7—N1—N2	114.0 (4)	C17—C16—H16	120.7
C7—N1—Zn1	126.1 (3)	C15—C16—H16	120.7
N2—N1—Zn1	119.4 (3)	C16—C17—C18	120.2 (5)
C8—N2—N1	112.8 (4)	C16—C17—H17	119.9
C8—N3—C9	128.7 (4)	C18—C17—H17	119.9
C8—N3—H3	114 (3)	C26—C18—C17	116.9 (4)
C9—N3—H3	116 (3)	C26—C18—C19	120.0 (5)
C15—N4—C26	118.1 (4)	C17—C18—C19	123.0 (5)
C15—N4—Zn1	129.6 (3)	C20—C19—C18	121.1 (5)
C26—N4—Zn1	112.1 (3)	C20—C19—H19	119.4
C24—N5—C25	118.6 (4)	C18—C19—H19	119.4
C24—N5—Zn1	128.2 (3)	C19—C20—C21	120.8 (5)
C25—N5—Zn1	113.2 (3)	C19—C20—H20	119.6
C27—N6—C31	115.6 (5)	C21—C20—H20	119.6
O1—C1—C2	118.5 (4)	C25—C21—C22	117.3 (5)
O1—C1—C6	123.8 (4)	C25—C21—C20	118.9 (5)
C2—C1—C6	117.7 (4)	C22—C21—C20	123.8 (5)
C3—C2—C1	122.0 (4)	C23—C22—C21	119.6 (5)
C3—C2—H2A	119.0	C23—C22—H22	120.2
C1—C2—H2A	119.0	C21—C22—H22	120.2
O2—C3—C2	122.1 (4)	C22—C23—C24	119.0 (5)
O2—C3—C4	118.2 (4)	C22—C23—H23	120.5
C2—C3—C4	119.7 (4)	C24—C23—H23	120.5
C5—C4—C3	119.5 (4)	N5—C24—C23	123.0 (5)
C5—C4—H4	120.2	N5—C24—H24	118.5
C3—C4—H4	120.2	C23—C24—H24	118.5
C4—C5—C6	123.3 (5)	N5—C25—C21	122.5 (4)
C4—C5—H5A	118.4	N5—C25—C26	117.8 (4)
C6—C5—H5A	118.4	C21—C25—C26	119.8 (4)
C5—C6—C1	117.7 (4)	N4—C26—C18	123.1 (4)
C5—C6—C7	118.3 (4)	N4—C26—C25	117.6 (4)
C1—C6—C7	123.9 (4)	C18—C26—C25	119.2 (4)
N1—C7—C6	124.6 (4)	N6—C27—C28	124.0 (5)
N1—C7—H7	117.7	N6—C27—H27	118.0
C6—C7—H7	117.7	C28—C27—H27	118.0
N2—C8—N3	117.0 (4)	C27—C28—C29	120.2 (5)
N2—C8—S1	128.2 (4)	C27—C28—H28	119.9
N3—C8—S1	114.7 (3)	C29—C28—H28	119.9
C10—C9—C14	119.4 (4)	C28—C29—C30	115.9 (5)
C10—C9—N3	123.7 (4)	C28—C29—C29^i^	122.6 (5)
C14—C9—N3	116.8 (4)	C30—C29—C29^i^	121.5 (5)
C9—C10—C11	119.6 (5)	C31—C30—C29	120.1 (5)
C9—C10—H10	120.2	C31—C30—H30	119.9
C11—C10—H10	120.2	C29—C30—H30	119.9
C10—C11—C12	120.6 (5)	N6—C31—C30	124.1 (5)
C10—C11—H11	119.7	N6—C31—H31	117.9
C12—C11—H11	119.7	C30—C31—H31	117.9
			
O1—Zn1—S1—C8	−90.8 (2)	C8—N3—C9—C10	33.2 (7)
N1—Zn1—S1—C8	−15.58 (18)	C8—N3—C9—C14	−151.2 (5)
N5—Zn1—S1—C8	85.89 (18)	C14—C9—C10—C11	−0.9 (7)
N4—Zn1—S1—C8	166.81 (18)	N3—C9—C10—C11	174.7 (4)
N1—Zn1—O1—C1	27.9 (4)	C9—C10—C11—C12	1.4 (7)
N5—Zn1—O1—C1	−75.3 (4)	C10—C11—C12—C13	−0.5 (8)
N4—Zn1—O1—C1	−153.8 (4)	C11—C12—C13—C14	−0.9 (8)
S1—Zn1—O1—C1	101.5 (4)	C12—C13—C14—C9	1.4 (7)
O1—Zn1—N1—C7	−18.6 (4)	C10—C9—C14—C13	−0.5 (7)
N5—Zn1—N1—C7	86.4 (4)	N3—C9—C14—C13	−176.3 (4)
S1—Zn1—N1—C7	−168.2 (4)	C26—N4—C15—C16	−1.2 (7)
O1—Zn1—N1—N2	169.6 (3)	Zn1—N4—C15—C16	173.0 (3)
N5—Zn1—N1—N2	−85.5 (3)	N4—C15—C16—C17	1.1 (7)
S1—Zn1—N1—N2	19.9 (3)	C15—C16—C17—C18	−0.5 (7)
C7—N1—N2—C8	172.2 (4)	C16—C17—C18—C26	0.2 (7)
Zn1—N1—N2—C8	−14.9 (5)	C16—C17—C18—C19	−178.2 (5)
O1—Zn1—N4—C15	−73.6 (4)	C26—C18—C19—C20	3.6 (7)
N5—Zn1—N4—C15	−178.8 (4)	C17—C18—C19—C20	−178.1 (5)
S1—Zn1—N4—C15	75.7 (4)	C18—C19—C20—C21	−3.3 (7)
O1—Zn1—N4—C26	101.0 (3)	C19—C20—C21—C25	0.3 (7)
N5—Zn1—N4—C26	−4.3 (3)	C19—C20—C21—C22	−177.2 (5)
S1—Zn1—N4—C26	−109.7 (3)	C25—C21—C22—C23	0.8 (7)
O1—Zn1—N5—C24	95.4 (4)	C20—C21—C22—C23	178.3 (4)
N1—Zn1—N5—C24	3.2 (4)	C21—C22—C23—C24	−1.5 (7)
N4—Zn1—N5—C24	−178.0 (4)	C25—N5—C24—C23	0.9 (7)
S1—Zn1—N5—C24	−82.8 (4)	Zn1—N5—C24—C23	−177.7 (3)
O1—Zn1—N5—C25	−83.3 (3)	C22—C23—C24—N5	0.7 (7)
N1—Zn1—N5—C25	−175.5 (3)	C24—N5—C25—C21	−1.7 (6)
N4—Zn1—N5—C25	3.3 (3)	Zn1—N5—C25—C21	177.1 (3)
S1—Zn1—N5—C25	98.5 (3)	C24—N5—C25—C26	179.2 (4)
Zn1—O1—C1—C2	156.0 (3)	Zn1—N5—C25—C26	−2.0 (5)
Zn1—O1—C1—C6	−24.9 (6)	C22—C21—C25—N5	0.9 (7)
O1—C1—C2—C3	−178.3 (4)	C20—C21—C25—N5	−176.8 (4)
C6—C1—C2—C3	2.5 (7)	C22—C21—C25—C26	180.0 (4)
C1—C2—C3—O2	178.1 (4)	C20—C21—C25—C26	2.3 (7)
C1—C2—C3—C4	−2.6 (8)	C15—N4—C26—C18	0.9 (6)
O2—C3—C4—C5	179.7 (5)	Zn1—N4—C26—C18	−174.4 (3)
C2—C3—C4—C5	0.3 (8)	C15—N4—C26—C25	179.8 (4)
C3—C4—C5—C6	2.0 (8)	Zn1—N4—C26—C25	4.6 (5)
C4—C5—C6—C1	−2.1 (8)	C17—C18—C26—N4	−0.3 (6)
C4—C5—C6—C7	176.5 (5)	C19—C18—C26—N4	178.0 (4)
O1—C1—C6—C5	−179.3 (4)	C17—C18—C26—C25	−179.3 (4)
C2—C1—C6—C5	−0.2 (7)	C19—C18—C26—C25	−0.9 (6)
O1—C1—C6—C7	2.2 (7)	N5—C25—C26—N4	−1.8 (6)
C2—C1—C6—C7	−178.7 (4)	C21—C25—C26—N4	179.0 (4)
N2—N1—C7—C6	177.8 (4)	N5—C25—C26—C18	177.2 (4)
Zn1—N1—C7—C6	5.5 (7)	C21—C25—C26—C18	−2.0 (6)
C5—C6—C7—N1	−170.8 (5)	C31—N6—C27—C28	0.2 (7)
C1—C6—C7—N1	7.7 (8)	N6—C27—C28—C29	0.8 (8)
N1—N2—C8—N3	177.6 (4)	C27—C28—C29—C30	−1.4 (7)
N1—N2—C8—S1	−4.2 (6)	C27—C28—C29—C29^i^	179.3 (5)
C9—N3—C8—N2	−2.7 (7)	C28—C29—C30—C31	1.1 (7)
C9—N3—C8—S1	178.8 (4)	C29^i^—C29—C30—C31	−179.6 (5)
Zn1—S1—C8—N2	16.5 (4)	C27—N6—C31—C30	−0.5 (7)
Zn1—S1—C8—N3	−165.2 (3)	C29—C30—C31—N6	−0.1 (8)

Symmetry codes: (i) −*x*, −*y*+2, −*z*+1.

### Hydrogen-bond geometry (Å, °)

**Table:**

*D*—H···*A*	*D*—H	H···*A*	*D*···*A*	*D*—H···*A*
O2—H2···N6	0.84 (5)	2.01 (5)	2.839 (6)	168 (6)
